# Cold Storage of 1- to 5-Day-Old Mummies and Newly Emerged Adults of *Aphelinus maculatus* for Biological Control of *Myzus persicae*

**DOI:** 10.3390/biology15201862

**Published:** 2026-10-16

**Authors:** Xiaoli Wang, Qingxian Liu, Rula Sa, Anqi Zhu, Wenhao Dai, Liqiong Yan, Liyan Zhang, Haiyan Jiang

**Affiliations:** 1 College of Forestry, Inner Mongolia Agricultural University, Hohhot 010019, China; nmwangxiaoli@163.com (X.W.); uqaz0813@163.com (A.Z.); liqiongyan@imau.edu.cn (L.Y.); zhangliyanyong@126.com (L.Z.); 2 National Field Observation and Research Station (Neimenggu Daxing’anling) for Forest Ecosystem, Genhe 022350, China; 3 Inner Mongolia Autonomous Region Forestry and Grassland Protection Station, Hohhot 010020, China; 17747356521@163.com; 4 Center for Biological Disaster Prevention and Control, National Forestry and Grassland Administration, Shenyang 110034, China; dwh311026@163.com

**Keywords:** *Aphelinus maculatus*, *Myzus persicae*, cold storage, parasitoid fitness, wolfberry

## Abstract

Production and field-release schedules are often asynchronous in augmentative biological control. We evaluated the cold-storage tolerance of 1- to 5-day-old mummies and newly emerged adults of *Aphelinus maculatus*, a parasitoid of *Myzus persicae*, at 4 and 8 °C. Adult emergence from stored mummies and survival of directly stored adults were recorded. Poststorage longevity, host-feeding capacity, parasitism capacity, offspring production, and offspring sex ratio were evaluated for selected cohorts stored at 8 °C. Storage at 8 °C generally resulted in higher mummy emergence and adult survival than storage at 4 °C. Among the tested mummy-age cohorts, 3-day-old mummies showed the highest emergence under the tested conditions. At 8 °C, emergence from this cohort was 90.0, 78.3, 64.7, 54.7, and 21.3% after 7, 14, 21, 30, and 60 d, respectively. Directly stored females survived better than males; female survival was 72.0% after 30 d and 20.3% at the 90-day storage endpoint. Increasing storage duration reduced poststorage longevity, host-feeding, parasitism, and offspring production. The female-to-male offspring sex ratio remained relatively stable during the 30 d poststorage assessment period. Under the tested conditions, storing 3-day-old mummies at 8 °C provides an option for delayed emergence, whereas direct adult storage at 8 °C may be useful for short-term holding.

## Simple Summary

Chinese wolfberry is an important crop for low-pesticide production because its fruits are widely used as food and medicinal products. The green peach aphid, *Myzus persicae*, can seriously damage wolfberry plants, whereas the parasitoid *Aphelinus maculatus* provides a potential biological control option. Reliable storage is needed to synchronize parasitoid production, transport, and field release. We evaluated the cold storage of 1- to 5-day-old mummy-age cohorts and newly emerged adults of *A. maculatus*. Storage at 8 °C generally resulted in higher mummy emergence and adult survival than storage at 4 °C. Among the tested mummy-age cohorts, 3-day-old mummies showed the highest emergence under the tested conditions. Directly stored females also showed higher survival than males, with 20.3% of females remaining viable at the 90-day storage endpoint. However, poststorage host-feeding, parasitism, and offspring production were evaluated only up to 30 d. These findings provide storage options for mass rearing, transport, and scheduled release of *A. maculatus* in wolfberry production.

## 1. Introduction

Chinese wolfberry, *Lycium* spp., is an important economic forest crop in northwestern China and is widely cultivated as a traditional medicinal material, functional food, and health product. The commercial value and sustainability of the wolfberry industry depend not only on yield but also on fruit quality and product safety [1]. The green peach aphid, *Myzus persicae* (Sulzer) (Hemiptera: Aphididae), is one of the major aphid pests associated with wolfberry [2]. It damages tender shoots, young leaves, flower buds, and fruiting branches through phloem feeding and also acts as an efficient vector of plant viruses, ultimately reducing photosynthetic capacity, plant vigor, and fruit quality. Because wolfberry products are directly consumed and are closely associated with health-related markets, repeated applications of chemical insecticides are undesirable due to pesticide residues, resistance development, and environmental risks [3,4]. Biological control using natural enemies is therefore an important component of sustainable pest management in wolfberry production systems [5].

*Aphelinus maculatus* Yasnosh (Hymenoptera: Aphelinidae) is an important parasitoid of *M. persicae*. Previous studies have shown that females suppress aphid populations not only through parasitism but also through host-feeding and ovipositor stabbing, which together increase aphid mortality and reduce host development and fecundity [6,7]. These biological traits indicate that *A. maculatus* has strong potential as a biological control agent, particularly in low-residue production systems. However, successful large-scale application of this parasitoid requires a reliable supply of high-quality individuals at the appropriate release time. In practice, field release is often constrained by the mismatch between continuous mass rearing and fluctuating field demand, as well as by unsuitable cold-storage techniques, uncertain storage duration, and variable poststorage quality, which may lead to inconsistent control efficacy. Cold storage is widely used to extend the shelf life of natural enemies, facilitate transport, and support scheduled releases in augmentative biological control programs [8,9,10]. Nevertheless, inappropriate cold-storage conditions can markedly reduce parasitoid survival, reproductive capacity, parasitism performance, and offspring quality [8,11]. For example, adults of *Trichogramma evanescens* can retain relatively high survival after short-term storage at 4 °C [12], whereas cold storage can significantly impair the survival, reproduction, and parasitism capacity of the whitefly parasitoids *Encarsia sophia* and *Eretmocerus hayati*, especially under prolonged exposure at 4 °C [11]. In *Pachycrepoideus vindemiae*, adults exhibit greater cold tolerance than pupal stages [13], while low-temperature storage in the *Telenomus remus* system can reduce progeny production and offspring survival and increase the proportion of male offspring [14]. These differences may be associated with species-specific physiological mechanisms, including diapause-related traits, antioxidant responses, cryoprotectant accumulation, and membrane lipid remodeling [8,15]. Therefore, cold-storage protocols cannot be generalized across parasitoid taxa and must be optimized according to the biological characteristics and application requirements of each natural enemy.

Temperature is a fundamental environmental factor regulating insect survival, development, reproduction, and species interactions. In biological control systems, understanding temperature-dependent responses is essential for improving parasitoid performance, storage, transport, and field deployment [16,17]. One major constraint in augmentative biological control is production-release asynchrony, in which laboratory or factory rearing proceeds continuously, whereas field demand changes with pest phenology, climate, crop growth stage, and management schedules. Cold storage can help bridge this mismatch by slowing development, extending storage duration, and increasing the flexibility of release programs [8,9,10]. However, cold exposure may also impose direct and sublethal stress, and its effects depend on storage temperature, storage duration, species, storage form, cohort age, and the physiological condition of insects before storage [8,15,18].

Cold-storage tolerance can vary among mummy-age cohorts and between immature and adult storage forms in parasitoids. In some species, immature stages tolerate cold storage better than adults, whereas in others adults or particular pupal stages are more suitable for storage [13,19,20]. In addition to direct mortality, cold storage can cause sublethal effects, including reduced longevity, impaired host-searching or host-feeding activity, lower fecundity, reduced parasitism, delayed development, and altered offspring sex ratios [11,21,22]. Prolonged cold exposure may also disrupt the synchrony between parasitoid emergence and host availability after release [23,24]. Thus, an effective storage protocol must preserve not only adult emergence or survival but also the poststorage biological performance that determines field efficacy.

The thermal development of *A. maculatus* has been studied previously [25], and its adult biological characteristics, host-feeding behavior, and ovipositor-stabbing effects on *M. persicae* have been documented [6,7]. However, information on cold storage of this parasitoid remains limited. In particular, no study has systematically compared the cold-storage responses of different mummy-age cohorts and newly emerged adults under the same storage conditions, and the poststorage effects on longevity, host-feeding, parasitism, offspring emergence, and offspring sex ratio remain insufficiently evaluated. This knowledge gap makes it difficult to determine suitable storage conditions for mass production, transport, and scheduled release of *A. maculatus*, thereby limiting its practical application in large-scale biological control of *M. persicae* in wolfberry systems.

To address this gap, we evaluated the cold storage of 1- to 5-day-old mummies and newly emerged adults (≤12 h old) of *A. maculatus* at 4 and 8 °C. These two temperatures were selected based on preliminary experiments conducted by our research group, in which mummies and adults of *A. maculatus* were stored at 2, 4, 6, and 8 °C for up to three months. The objectives of this study were to (1) compare cold-storage tolerance among mummy-age cohorts and between sexes of newly emerged adults, (2) identify suitable storage temperatures and durations for each storage form, and (3) evaluate poststorage effects on longevity, host-feeding, parasitism, offspring emergence, and offspring sex ratio. We expected that storage suitability would differ between mummy and adult storage and that an intermediate mummy age would provide the best balance between adult emergence and poststorage performance.

## 2. Materials and Methods

### 2.1. Insect Rearing

The foundational colony of *A. maculatus* was established from mummified *M. persicae* collected in Chinese wolfberry (*Lycium chinense* Mill.) plantations at the Science and Technology Park of Inner Mongolia Agricultural University, Hohhot, China. Parasitoids and aphid hosts were maintained at 26 ± 1 °C, 60–70% relative humidity (RH), and a 16:8 h light:dark photoperiod (RQH-450, Shanghai Yiheng Scientific Instrument Co., Ltd., Shanghai, Shanghai, China). The aphid host, *M. persicae*, was continuously reared on potted Chinese cabbage, *Brassica campestris* L. cv. ‘Shanghai Qing’ (Jinshengda Seed Company, Nanjing, Jiangsu, China), under the same environmental conditions [6,25].

### 2.2. Preparation of Experimental Cohorts

#### 2.2.1. Mummy-Age Cohorts

To obtain mummy-age cohorts of known and uniform age, apterous adult *M. persicae* were introduced onto potted *B. campestris* seedlings and allowed to establish aphid colonies. Newly emerged *A. maculatus* adults were then released into cages (laboratory-made) for parasitization. Mummy formation was checked daily. During the first 6 days after parasitoid exposure, all newly formed mummies were removed and discarded because their exact time of mummification was uncertain [25]. Beginning on day 7 after parasitoid exposure, newly formed black mummies were collected every 24 h. The day of visible mummy formation was defined as 0 days after mummification. The collected mummies were maintained under standard laboratory conditions until they reached 1-, 2-, 3-, 4-, or 5 days after mummification and were assigned to the corresponding mummy-age cohorts.

Thus, mummy age was defined strictly by the time elapsed since visible mummification. The internal developmental condition of the parasitoid inside each mummy was not examined by dissection, histology, or physiological assays. Accordingly, the terms 1-, 2-, 3-, 4-, and 5-day-old mummies refer only to mummy-age cohorts and do not indicate verified internal developmental stages. Each treatment consisted of three independent biological replicates, with 100 mummies per replicate. Before cold storage, mummies were placed in Petri dishes (BKMAMLAB, Changsha, Hunan, China) lined with moistened filter paper.

#### 2.2.2. Adult Storage Cohorts

A separate group of mummies was maintained at 26 ± 1 °C until adult emergence. Newly emerged adults, ≤12 h old, were collected, sexed, and grouped into mixed-sex cohorts at a female:male ratio of 1:1. Mixed-sex grouping provided adults from all treatments with comparable opportunities for mating before and during holding or cold storage and minimized differences in mating opportunity among treatments. Unstored adult controls were maintained under the same mixed-sex conditions.

Adults were continuously supplied with a 10% *w*/*v* aqueous sucrose (Sinopharm Chemical Reagent Co., Ltd., Tianjin, Tianjin, China) solution through sterile cotton wicks during holding and cold storage. The sucrose-saturated wicks served as the food and moisture source and were replaced three times per week. Unstored adult controls received the same sucrose supply under identical laboratory conditions [9].

Mating status was not treated as an independent experimental factor, and the mating status of individual adults was not verified. Therefore, this experiment was not designed to compare mated and unmated adults. Instead, sex ratio, rearing conditions, sucrose supply, storage procedures, and poststorage handling were standardized across adult-storage treatments. This design minimized differences in mating opportunity, although individual variation in mating status could not be completely excluded.

For poststorage biological-performance assays, one surviving female and one surviving male from the same storage treatment were paired using the same procedure for all treatments. Each pair was provided with the same opportunity for mating before and during the bioassay.

### 2.3. Cold-Storage Treatments

Preliminary experiments conducted by our research group evaluated the cold-storage responses of *A. maculatus* mummies and adults at 2, 4, 6, and 8 °C for up to 3 months [26]. Based on these preliminary observations and previous information on temperature-dependent responses in parasitoids, 4 °C and 8 °C were selected for detailed evaluation because they represented two operationally relevant storage temperatures with different levels of chilling intensity.

For mummy storage, 1-, 2-, 3-, 4-, and 5-day-old mummy-age cohorts were stored at 4 °C or 8 °C for 7, 14, 21, 30, or 60 days. For adult storage, newly emerged adults, ≤12 h old, were stored at 4 °C or 8 °C for 7, 14, 21, 30, 60, or 90 days. Female and male adults were exposed to the same storage temperatures and durations, and survival was analyzed separately by sex.

The 90 d adult-storage treatment was included to determine the upper survival limit of directly stored adults under the tested low-temperature holding conditions. It was not intended to estimate the normal adult lifespan of *A. maculatus* under standard rearing conditions or to represent a recommended field-release interval. At 8 °C, reduced metabolic activity and energy expenditure may allow some adults to remain viable for an extended period compared with actively feeding and reproducing adults maintained at 26 °C. In addition, adults were continuously supplied with a 10% *w*/*v* sucrose solution via sterile cotton wicks during storage to reduce starvation-related mortality. Nevertheless, survival at the storage endpoint does not indicate that adults maintained normal host-feeding, parasitism, or reproductive performance throughout the storage period.

During cold storage, adults were continuously supplied with a 10% *w*/*v* sucrose solution via sterile cotton wicks. After cold storage, all samples were rewarmed stepwise to reduce thermal shock. Samples were first transferred from the cold-storage chamber (RQH-450, Shanghai Yiheng Scientific Instrument Co., Ltd., Shanghai, Shanghai, China) to 12 ± 1 °C for 2 h and then moved to 26 ± 1 °C for recovery and subsequent assessment. For directly stored adults, survival was assessed after rewarming. An adult was considered alive if it resumed coordinated locomotor activity within 1 h at 26 ± 1 °C [27]. Unstored mummies and newly emerged adults maintained continuously at 26 ± 1 °C, 60–70% RH, and a 16:8 h light:dark photoperiod served as controls. Detailed experimental layouts and response variables are shown in Table 1.

### 2.4. Experimental Design and Assessment of Poststorage Biological Performance at 8 °C

Based on the screening assays described in Section 2.2 and Section 2.3, poststorage biological performance was evaluated at 8 °C using two storage strategies: adults emerging from stored 3-day-old mummy-age cohorts and directly stored newly emerged adults. The 8 °C treatment was selected because the preceding storage assays showed higher mummy emergence and adult survival at this temperature than at 4 °C. Among the five mummy-age cohorts examined under the same screening conditions, the 3-day-old cohort was selected as the representative mummy-storage cohort because it showed the most favorable emergence response across the tested storage durations. This selection was based solely on mummy-age-associated emergence performance and does not imply a confirmed internal developmental stage.

Poststorage biological performance was assessed after 7, 14, 21, and 30 days of storage, together with an unstored control. These intervals were selected to represent short- and medium-term holding conditions under the more promising temperature identified in the screening assays. The functional assessment was designed to compare the selected mummy-storage cohort with directly stored adults under standardized 8 °C conditions; it was not intended to compare the poststorage performance of all mummy-age cohorts and storage temperatures.

Each replicate consisted of one surviving female and one surviving male from the same storage treatment. Each pair was placed in a leaf-rearing chamber (laboratory-made) containing 50 healthy second- to third-instar *M. persicae* (Figure 1). The same pairing and bioassay procedures were used for all treatments to standardize mating opportunity. Because the mating status of individual adults was not verified, the assay was not designed to compare mated and unmated adults.

Adult survival was recorded every 24 h. Adult longevity was defined as the number of days from the beginning of the poststorage bioassay until death. An adult was considered dead when it failed to resume coordinated movement after gentle stimulation.

Each surviving female was provided with a fresh group of 50 s- to third-instar aphids every 24 h throughout her lifetime. Removed aphids were maintained separately under standard laboratory conditions and examined for mortality and mummy formation. Aphids that died without subsequently forming mummies were recorded as host-feeding mortality, whereas aphids that developed into black mummies were recorded as successfully parasitized. Lifetime host-feeding capacity and parasitism capacity were calculated as the cumulative numbers of host-feeding mortalities and mummies, respectively, produced by each female during the bioassay.

All mummies produced by each female were maintained at 26 ± 1 °C, 60–70% relative humidity, and a 16:8 h light:dark photoperiod until parasitoid emergence ceased. Offspring production was recorded as the total number of emerged parasitoids per female. Offspring emergence rate was calculated as follows:

(1) $$\begin{array}{c} Offspring\;emergence\;rate=\frac{\mathrm{number}\;\mathrm{of}\;\mathrm{emerged}\;\mathrm{parasitoids}}{\mathrm{number}\;\mathrm{of}\;\mathrm{mummies}\;\mathrm{produced}}\times 100. \end{array}$$

Emerged offspring were sexed under a stereomicroscope. The proportion of female offspring was calculated as follows:

(2) $$\begin{array}{c} Female\;offspring\;proportion=\frac{\mathrm{number}\;\mathrm{of}\;\mathrm{emerged}\;\mathrm{females}}{\mathrm{total}\;\mathrm{number}\;\mathrm{of}\;\mathrm{emerged}\;\mathrm{offspring}}\times 100. \end{array}$$

Replicates without emerged offspring were excluded from the offspring sex-ratio analysis.

### 2.5. Statistical Analysis

All analyses were performed in R version 4.6.1 (R Core Team, R Foundation for Statistical Computing, Vienna, Austria; [https://www.r-project.org/], accessed on 20 May 2026). Adult emergence from stored mummies was analyzed using a binomial generalized linear model, GLM, based on the numbers of emerged and non-emerged individuals. Storage temperature, storage duration, mummy age, and their interactions were included as fixed effects.

Survival of directly stored adults was analyzed using a binomial GLM based on the numbers of surviving and dead adults, with storage temperature, storage duration, sex, and their interactions as fixed effects.

Poststorage adult longevity was analyzed using a linear mixed-effects model, LMM, with storage strategy, storage duration, sex, and their interactions as fixed effects and mating-pair identity as a random effect. Lifetime host-feeding capacity and parasitism capacity were analyzed separately using LMMs, with storage strategy, storage duration, and their interaction as fixed effects.

Offspring production was analyzed using a negative binomial GLM based on the total number of emerged offspring per female. Offspring emergence rate was analyzed using a binomial GLM based on the numbers of emerged and non-emerged offspring. The proportion of female offspring was analyzed using a binomial GLM based on the numbers of female and male offspring. Storage strategy, storage duration, and their interaction were included as fixed effects in these poststorage analyses.

When significant interactions were detected, simple-effect comparisons were performed using estimated marginal means with Tukey adjustment. Model assumptions and overdispersion were assessed using residual diagnostics. The significance level was set at α = 0.05. Adult emergence, adult survival, offspring emergence rate, and offspring sex ratio were analyzed as endpoint count or proportion data.

## 3. Results

### 3.1. Adult Emergence from Stored Mummy-Age Cohorts

Adult emergence from stored *A. maculatus* mummies was significantly affected by storage temperature, storage duration, mummy age, and their interactions (GLM, *p* < 0.001; Figure 2).

Adult emergence after storage at 8 °C was higher than that after storage at 4 °C across the tested mummy-age cohorts and storage durations. The adult emergence of the unstored control was 93.0%. At 4 °C, adult emergence decreased with increasing storage duration. After 30 d of storage, emergence ranged from 1.3% to 5.7% among the mummy-age cohorts, and no adults emerged after 60 d of storage. At 8 °C, adult emergence also decreased with increasing storage duration. Among the five mummy-age cohorts, 3-day-old mummies had emergence values of 90.0%, 78.3%, 64.7%, 54.7%, and 21.3% after 7, 14, 21, 30, and 60 d of storage, respectively.

### 3.2. Survival of Directly Stored Adults

Storage temperature, storage duration, and sex significantly affected the survival of directly stored adults (Figure 3; GLM likelihood-ratio tests: temperature, χ^2^ = 2492.85, *df* = 1, *p* < 0.001; duration, χ^2^ = 1785.90, *df* = 5, *p* < 0.001; sex, χ^2^ = 284.08, *df* = 1, *p* < 0.001). In the unstored adult control, survival was 100% for both females and males. At 4 °C, male survival reached 0% after 30 d of storage, whereas female survival reached 0% after 60 d. At 8 °C, female survival was 97.0%, 72.0%, and 20.3% after 7, 30, and 90 d of storage, respectively. Male survival at 8 °C decreased with storage duration and reached 0% after 90 d.

### 3.3. Poststorage Biological Performance at 8 °C

#### 3.3.1. Adult Longevity

Poststorage adult longevity was significantly affected by storage strategy, storage duration, sex, and their interactions (Figure 4). For adults emerging from stored 3-day-old mummy-age cohorts, female longevity decreased from 39.8 d in the unstored control to 20.1 d after 30 d of storage. Male longevity also decreased with increasing storage duration.

For directly stored adults, female longevity ranged from 35.4 to 39.8 d across storage durations from 0 to 30 d, and male longevity ranged from 19.3 to 21.3 d. No significant difference in longevity was detected among storage durations for directly stored adults within this storage interval (*p* = 0.865).

#### 3.3.2. Host-Feeding and Parasitism Capacities

Storage strategy, storage duration, and their interaction significantly affected lifetime host-feeding capacity and lifetime parasitism capacity (Figure 5). In adults emerging from stored 3-day-old mummy-age cohorts, host-feeding capacity decreased from 96.5 aphids per female in the unstored control to 40.8 aphids per female after 30 days of storage. In directly stored adults, host-feeding capacity changed from 96.5 to 78.8 aphids per female over the same storage interval. Parasitism capacity in adults emerging from stored 3-day-old mummy-age cohorts decreased from 463.8 to 131.5 mummies per female between the unstored control and 30 days of storage. In directly stored adults, parasitism capacity changed from 463.8 to 271.7 mummies per female over the same interval.

#### 3.3.3. Offspring Production and Sex Ratio

Offspring production decreased with increasing storage duration in both storage strategies (Figure 6). In the mummy-derived cohort, the mean number of emerged offspring decreased from 458.5 individuals per female in the unstored control to 236.9, 202.6, 136.5, and 124.6 individuals per female after 7, 14, 21, and 30 days of storage, respectively. In the directly stored adult cohort, offspring production decreased from 458.5 individuals per female in the control to 444.0, 300.9, 267.1, and 260.5 individuals per female after 7, 14, 21, and 30 days of storage, respectively.

Offspring sex ratio was expressed as the female-to-male ratio. In the directly stored adult cohort, the ratio was 1.03 in the control and 1.03, 1.03, 1.04, and 1.03 after 7, 14, 21, and 30 days of storage, respectively. In the mummy-derived cohort, the ratio was 1.03 in the control and decreased to 0.98 after 30 days of storage.

## 4. Discussion

This study provides a comparative assessment of cold storage in five mummy-age cohorts and newly emerged adults of *Aphelinus maculatus*. Storage outcomes were affected by storage temperature, storage duration, mummy age, and sex in directly stored adults. Storage at 8 °C resulted in higher adult emergence from stored mummies and higher adult survival than storage at 4 °C. Among the tested mummy-age cohorts, 3-day-old mummies showed the highest emergence under the two temperatures evaluated in this study, 4 °C and 8 °C. At 8 °C, emergence from this cohort remained at 54.7% after 30 days of storage. Directly stored females also showed higher survival than males. However, increasing storage duration reduced poststorage longevity, host-feeding capacity, parasitism capacity, and offspring production. These findings indicate that storage temperature, duration, storage form, and mummy age should be considered together when developing storage protocols for *A. maculatus*. Because the internal developmental condition of parasitoids inside the mummies was not verified, the results should not be interpreted as evidence that 3-day-old mummies represent a specific internal developmental stage with universally superior cold tolerance.

Mummy age in this study was defined as the number of days elapsed since visible mummification. The internal developmental condition of the parasitoid inside the mummy was not examined by dissection, histology, or physiological assays. Therefore, the 1-, 2-, 3-, 4-, and 5-day-old treatments represent mummy-age cohorts rather than verified internal developmental stages. Previous studies have shown that thermal responses may vary with developmental timing, thermal history, energy allocation, and physiological condition [13,19]. Previous work on the thermal development of *A. maculatus* parasitizing *Myzus persicae* also highlights the importance of developmental rate and temperature in this parasitoid-host system [20]. These findings provide biological context for the differences observed among mummy-age cohorts, but they do not identify the internal developmental state corresponding to the 3-day-old cohort. Anatomical and physiological studies across successive mummy ages are therefore needed to determine whether the observed emergence pattern is associated with a particular internal condition and to clarify its relationship with cold tolerance and poststorage quality.

Sex-specific survival patterns were evident in directly stored adults. Female survival was higher than male survival under the same storage conditions, particularly at 8 °C. At 8 °C, female survival remained 72.0% after 30 days and 20.3% at the 90-day storage endpoint, whereas male survival declined to zero by 90 days. Sex differences in cold tolerance have also been reported in other parasitoids and may be associated with differences in body size, energetic reserves, stress tolerance, and reproductive allocation [11,28,29]. From an applied perspective, higher female survival is beneficial because females directly contribute to parasitism and host-feeding. However, reduced male survival may limit mating opportunities and fertilization success after storage. Therefore, survival at the storage endpoint should not be interpreted as evidence of sustained reproductive performance during the entire storage period.

Poststorage biological performance is an important indicator of parasitoid quality. Survival or emergence alone does not guarantee field efficacy because parasitoids must locate hosts, feed, oviposit, and produce viable offspring after release. In the present study, host-feeding capacity and parasitism capacity decreased with increasing storage duration in both storage strategies. Adults emerging from stored mummies showed lower poststorage host-feeding and parasitism capacities after longer storage, whereas directly stored adults retained higher short-term performance. These findings indicate that direct adult storage may be useful when parasitoids are needed for immediate release, whereas mummy storage may be more suitable when delayed emergence, transport convenience, or production flexibility is required.

Cold-induced reductions in parasitism may result from several nonexclusive mechanisms. Prolonged chilling can deplete energy reserves, impair neuromuscular function, reduce host-searching ability, and alter reproductive physiology [8,18]. Because host-feeding and parasitism require active movement, host recognition, probing, and oviposition, these behaviors may be more sensitive to sublethal cold stress than simple survival. The observed decline in host-feeding and parasitism capacity therefore emphasizes the importance of measuring behavioral and reproductive traits when evaluating storage protocols for biological control agents.

Offspring production declined with increasing storage duration in both storage strategies. This suggests that once parasitism was successful, offspring development was less affected than maternal host attack. A similar distinction between maternal performance and offspring developmental success has been observed in other cold-stored parasitoids [11,21,22]. For mass production, this result is encouraging because short- to medium-term storage may preserve offspring viability, even though the number of hosts parasitized by each female declines.

Prolonged storage also shifted offspring sex allocation toward males. In haplodiploid parasitoids, fertilized eggs develop into females and unfertilized eggs develop into males; therefore, male-biased offspring can reflect reduced sperm availability, reduced sperm viability, impaired fertilization, or altered maternal allocation after stress [28,30]. Similar male-biased shifts after cold storage have been reported in other parasitoids [31,32]. For biological control, female-biased or balanced offspring production is generally preferred because females are responsible for host suppression. In the present study, directly stored adults maintained near-parity or slightly female-biased offspring consistently up to 30 d. In contrast, adults emerging from 3-day-old mummies suffered a significant reduction in the proportion of female offspring after 30 days of storage. This indicates that 30 d represents a critical operational threshold: for mummy storage, extending beyond 30 days compromises demographic quality through a reduced female proportion, whereas for direct adult storage, 30 days marks the safe practical window before male mortality and potential mating constraints become pronounced.

These findings have direct implications for biological control of *M. persicae* in wolfberry. Because wolfberry is used as a medicinal and health food product, reducing chemical insecticide input is an important management goal. *Aphelinus maculatus* can suppress aphids through parasitism, host-feeding, and ovipositor stabbing [6,7], but its practical value depends on whether sufficient high-quality parasitoids can be supplied at the right time. The storage protocol identified here can help bridge the mismatch between continuous laboratory production and fluctuating field demand. Stored 3-day-old mummies may be useful for transport and scheduled releases, whereas newly emerged adults may be held briefly before release when immediate aphid suppression is needed. Thus, cold storage at 8 °C can improve the operational flexibility of *A. maculatus*-based biological control programs.

This study has several inherent limitations that should be addressed in future work. First, cold storage treatments were evaluated under constant thermal regimes in the laboratory, whereas commercial shipping and field environments involve fluctuating temperatures and microclimates that can alter cold injury and recovery dynamics [19,27]. Additionally, long-term laboratory rearing of the colony (32 generations) may have modified cold hardiness relative to wild populations [33,34]. Second, poststorage performance was evaluated using *Brassica campestris* as the rearing substrate rather than the target host plant, Chinese wolfberry (*Lycium* spp.); substantial differences in plant architecture, leaf surface texture, and phytochemical profiles between *Brassica* and *Lycium* could influence parasitoid foraging efficiency and host-suppression efficacy in field scenarios. Third, while key demographic and behavioral traits were systematically quantified, the underlying physiological and molecular mechanisms—such as energy reserve depletion, cryoprotectant accumulation, antioxidant responses, and related gene expression—were not deeply investigated in this study.

Future research should focus on evaluating fluctuating temperature regimes, pre-storage thermal acclimation, and commercial transport conditions to optimize practical storage protocols. Field and semi-field trials in wolfberry orchards are necessary to confirm whether laboratory performance translates directly to effective field pest suppression. Furthermore, comparative analyses between laboratory and wild populations, combined with in-depth physiological and metabolic profiling, will provide a mechanistic understanding of thermal tolerance and help refine mass-rearing and release programs for *A. maculatus*.

## 5. Conclusions

Storage at 8 °C was more suitable than storage at 4 °C for the tested storage forms of *Aphelinus maculatus*. Among the tested mummy-age cohorts, 3-day-old mummies showed the highest emergence under the two temperatures evaluated in this study, 4 °C and 8 °C. At 8 °C, this cohort retained 54.7% emergence after 30 days of storage and therefore represents an operational option when delayed emergence, transport convenience, or scheduled release is required.

Direct storage of newly emerged adults at 8 °C showed a clear survival advantage, particularly for females. Female survival was 72.0% after 30 days and 20.3% at the 90-day storage endpoint, whereas male survival declined to zero by 90 days. Thus, direct adult storage at 8 °C may provide greater survival-based storage flexibility than mummy storage. However, poststorage host-feeding capacity, parasitism capacity, offspring production, and offspring sex ratio were evaluated only up to 30 days. Therefore, survival at 90 days should not be interpreted as evidence of sustained reproductive fitness or field efficacy beyond 30 days.

Under the tested conditions, direct adult storage at 8 °C is suitable for short-term holding and immediate release, whereas storage of 3-day-old mummies at 8 °C provides an option for delayed emergence and scheduled release. Further studies should evaluate reproductive performance after 60- and 90-day adult storage, as well as field efficacy under fluctuating temperature and wolfberry-production conditions.

## Figures and Tables

**Figure 1:**
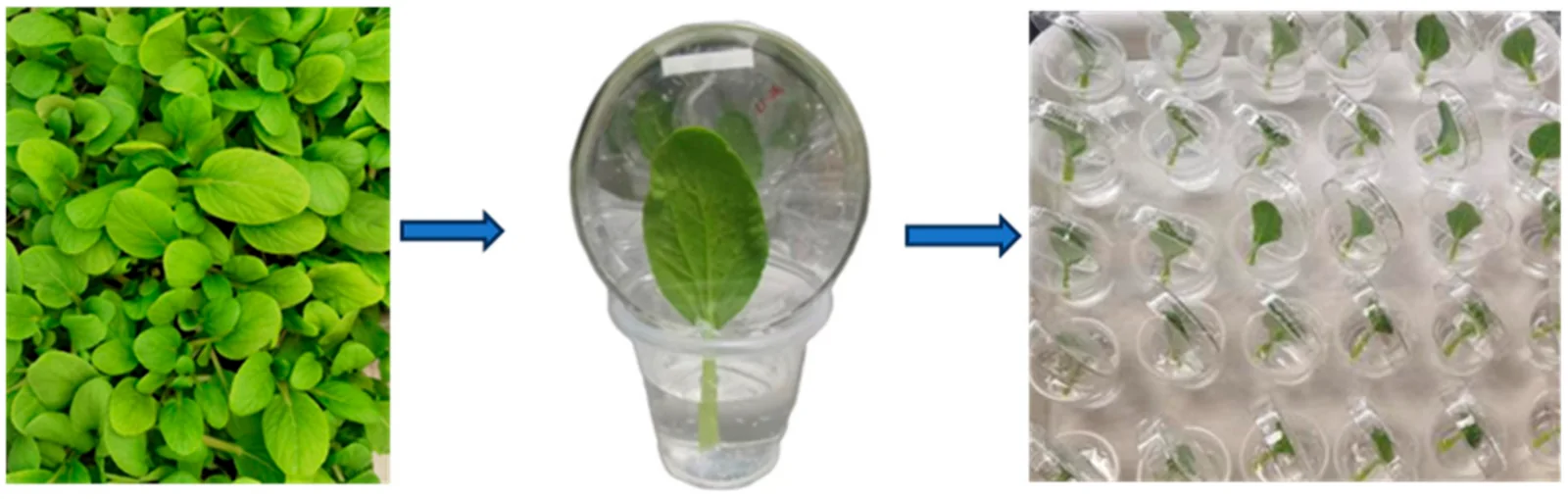
Leaf-rearing chamber used for poststorage assessment of *Aphelinus maculatus*. Young *Brassica campestris* leaves were placed abaxial side up in Petri dishes, with the petioles extending outside the dish. The dishes were sealed with plastic wrap, perforated with insect pins for ventilation, and placed over cups of water to keep the petioles submerged and maintain leaf turgor.

**Figure 2:**
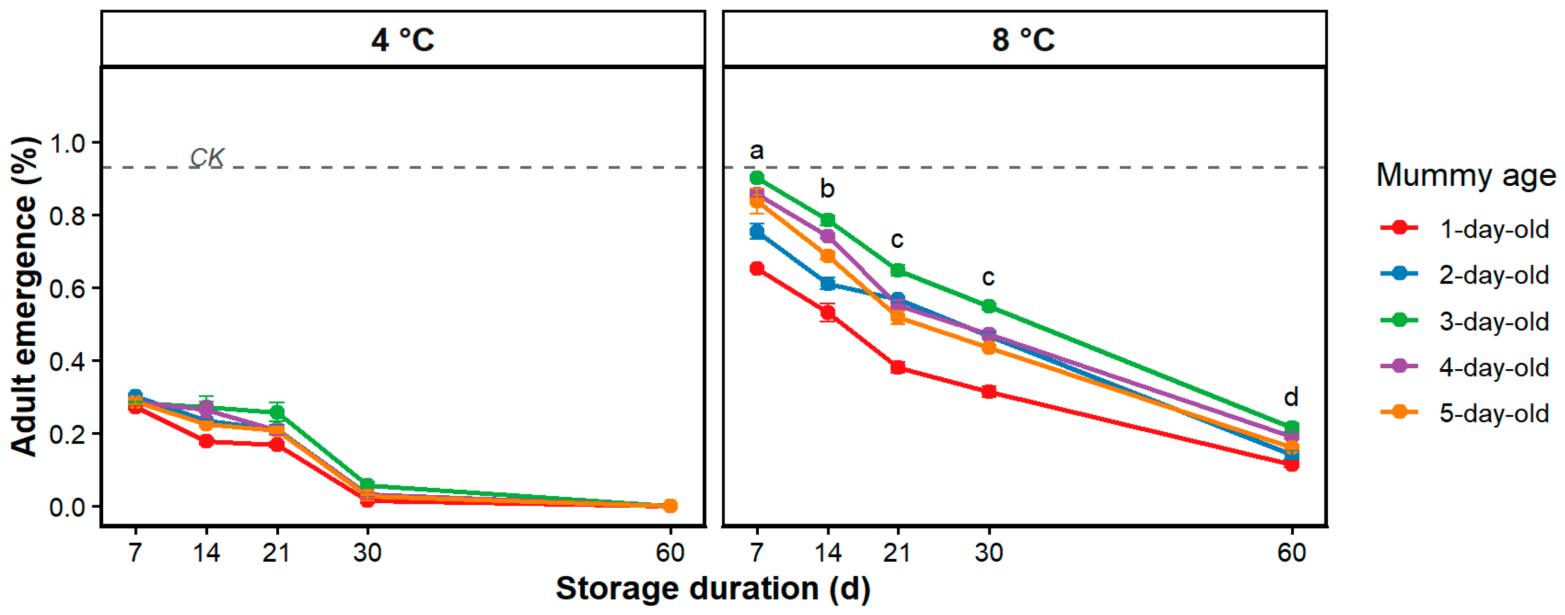
Adult emergence (%) of *A. maculatus* from 1- to 5-day-old mummy-age cohorts after cold storage at 4 °C and 8 °C for different durations. CK represents the unstored mummy control maintained at 26 ± 1 °C. The dashed line indicates the adult emergence rate of the unstored mummy control. Values are presented as mean ± SD. Different lowercase letters indicate significant differences among storage durations within the same mummy-age cohort and temperature treatment, *p* < 0.05.

**Figure 3:**
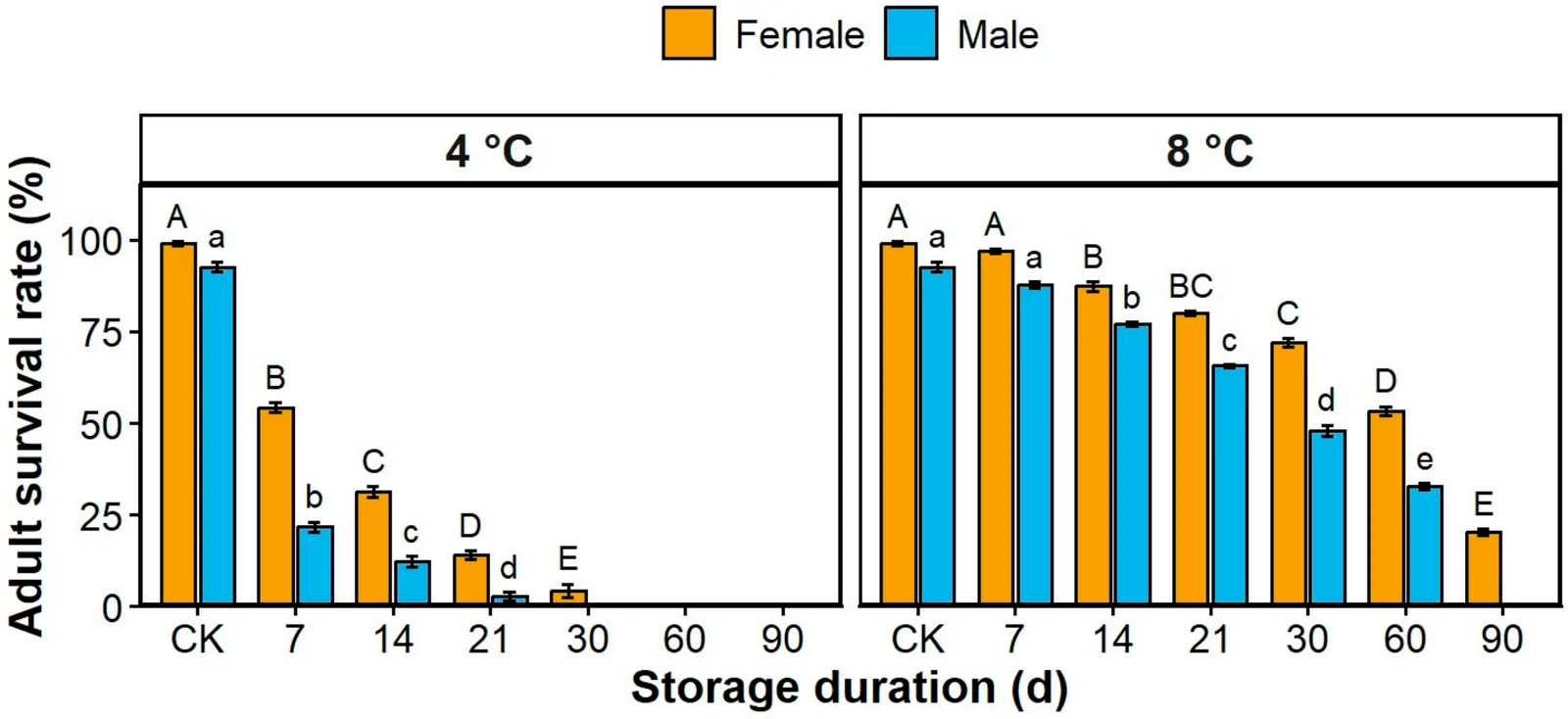
Survival (%) of directly stored adult *A. maculatus* females and males after cold storage at 4 °C and 8 °C for different durations. CK represents the unstored adult control maintained at 26 ± 1 °C. Values are presented as mean ± SE. Under the same temperature condition, different lowercase letters indicate significant differences among storage durations for males, whereas different uppercase letters indicate significant differences among storage durations for females, *p* < 0.05.

**Figure 4:**
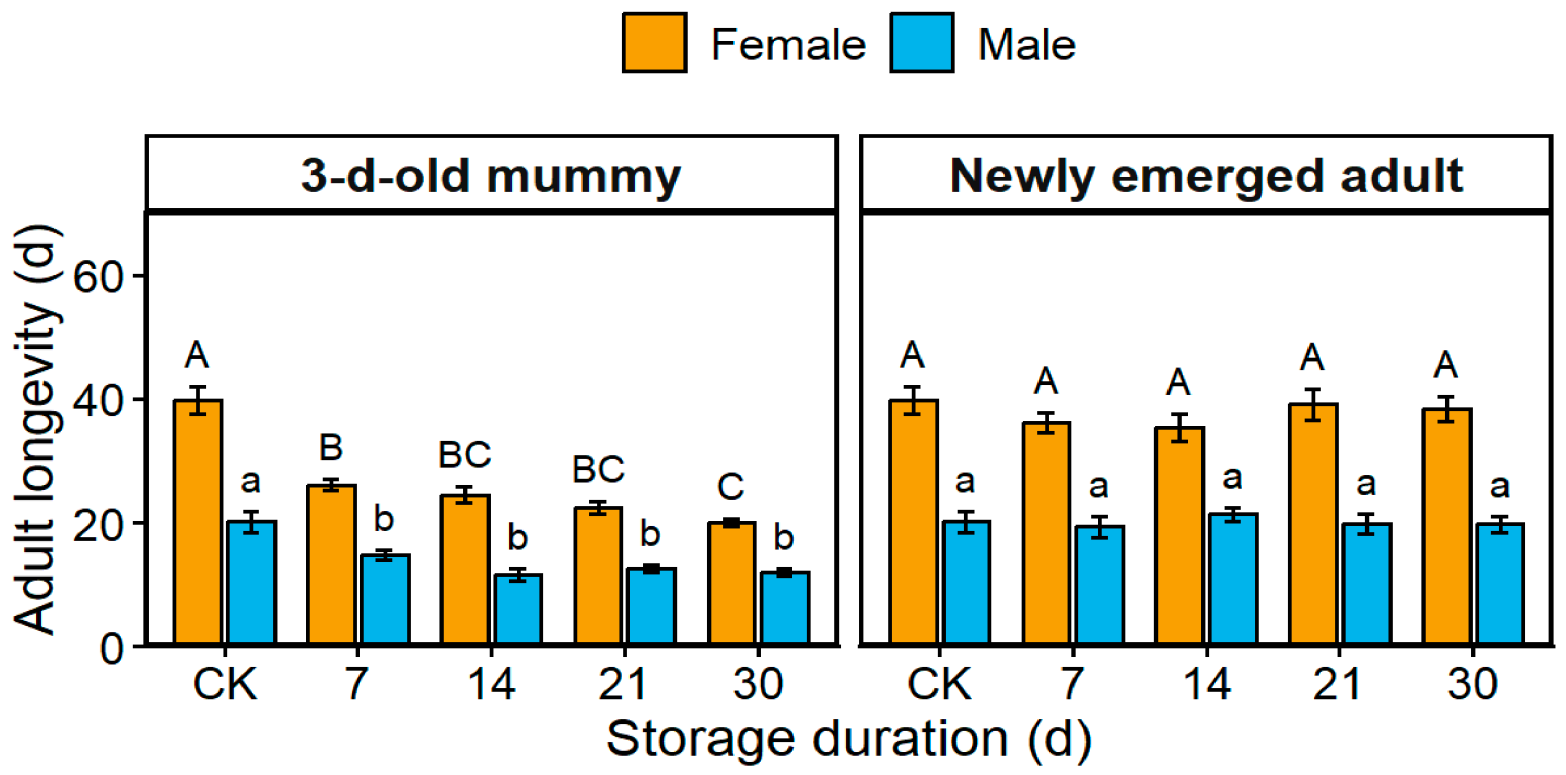
Poststorage longevity of *A. maculatus* adults after cold storage at 8 °C. (**Left**): adults emerging from stored 3-day-old mummy-age cohorts; (**right**): directly stored newly emerged adults. Values are presented as mean ± SE. Within the same storage strategy, different lowercase letters indicate significant differences among storage durations in males, whereas different uppercase letters indicate significant differences among storage durations in females, *p* < 0.05.

**Figure 5:**
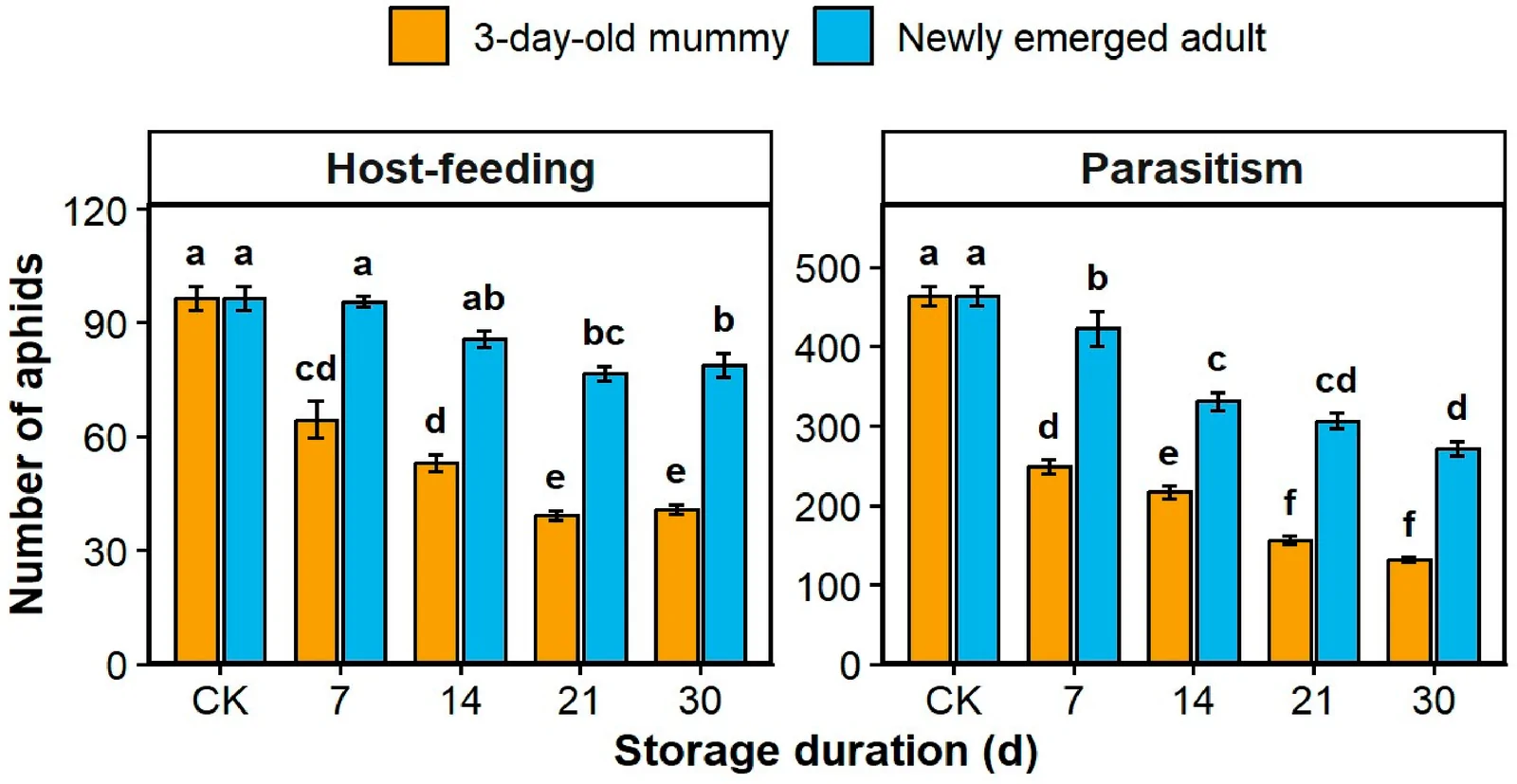
Lifetime host-feeding and parasitism capacities of *A. maculatus* females after cold storage at 8 °C. Values are presented as mean ± SE. Different lowercase letters indicate significant differences among storage strategy and storage-duration treatments, *p* < 0.05.

**Figure 6:**
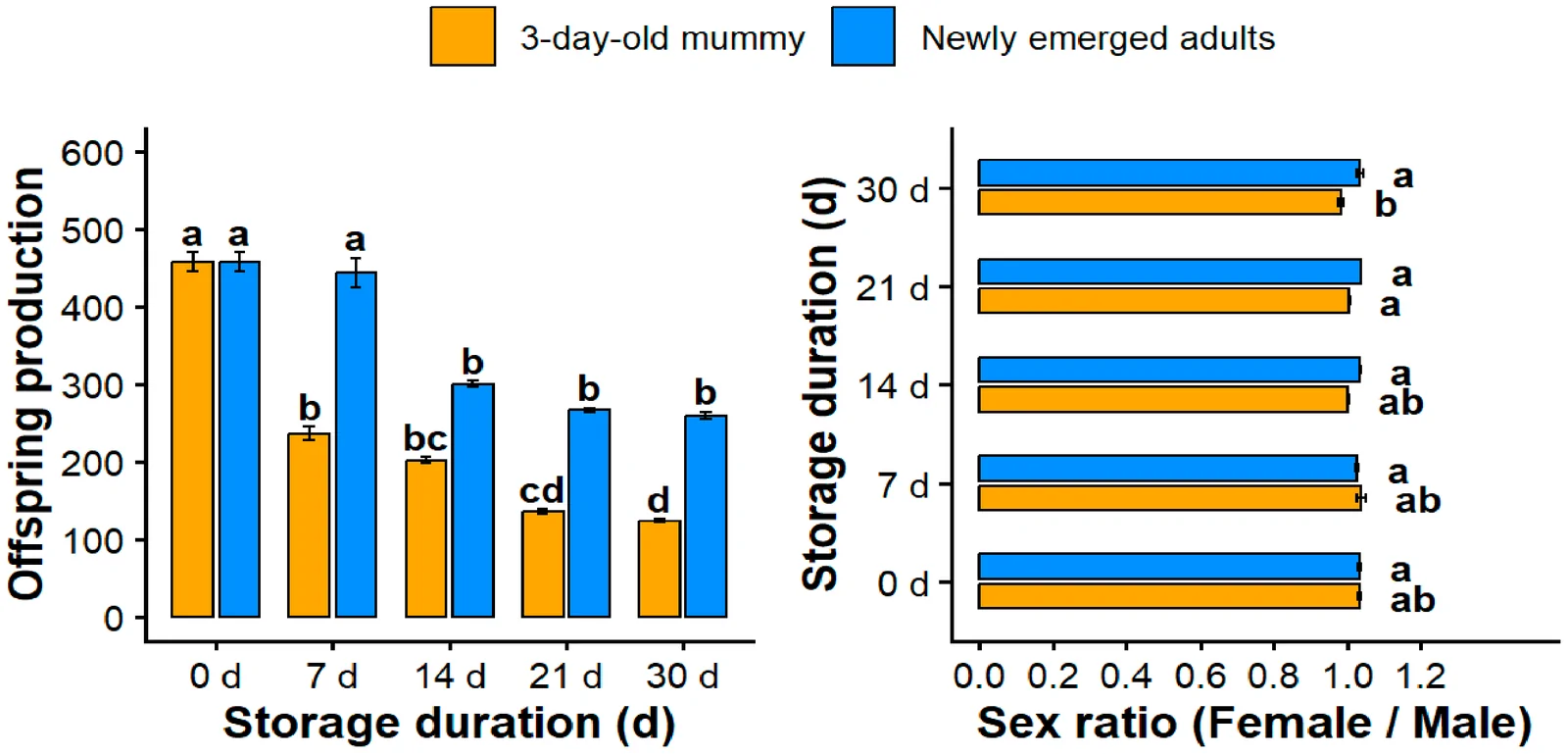
Offspring production and offspring sex ratios of *A. maculatus* after storage at 8 °C. Offspring production is presented as the mean number of emerged offspring per female, and offspring sex ratio is expressed as the female-to-male ratio. Values are presented as mean ± SE. Different lowercase letters indicate significant differences among storage strategy and storage-duration treatments, *p* < 0.05.

**Table 1:** Experimental design and response variables for cold storage assays of *A. maculatus*.

Experiment	Storage Temperature (°C)	Storage Duration (d)	Storage Form/Cohort	Individuals per Replicate	Replicates	Response Variable
Control (Mummy)	26 ± 1	0	Unstored mummies	100	3	Adult emergence rate
Control (Unstored adult)	26 ± 1	0	Newly emerged adults, ≤12 h old	100 (50 females + 50 males)	3	Adult survival rate
Mummy storage	4, 8	7, 14, 21, 30, 60	1–5-day-old mummy-age cohorts	100	3	Adult emergence rate
Adult storage	4, 8	7, 14, 21, 30, 60, 90	Newly emerged adults, ≤12 h old	100 (50 females + 50 males)	3	Adult survival rate

Note: Adult emergence rate = (number of emerged adults/number of stored mummies) × 100. Adult survival rate = (number of surviving adults/number of stored adults) × 100. During adult holding and cold storage, adults were continuously provided with a 10% *w*/*v* aqueous sucrose solution through sterile cotton wicks. The sucrose-saturated cotton wicks served as both the food and moisture source and were refreshed three times per week. The unstored adult control group was maintained at 26 ± 1 °C under the same humidity, photoperiod, sex ratio, and sucrose-supply conditions as the cold-storage treatments.

## Data Availability

The original contributions presented in this study are included in the article. Further inquiries can be directed to the corresponding authors.
